# Role of P2X7 Receptors in Immune Responses During Neurodegeneration

**DOI:** 10.3389/fncel.2021.662935

**Published:** 2021-05-26

**Authors:** Ágatha Oliveira-Giacomelli, Lyvia Lintzmaier Petiz, Roberta Andrejew, Natalia Turrini, Jean Bezerra Silva, Ulrich Sack, Henning Ulrich

**Affiliations:** ^1^Department of Biochemistry, Institute of Chemistry, University of São Paulo, São Paulo, Brazil; ^2^Institute of Clinical Immunology, Medical Faculty, University of Leipzig, Leipzig, Germany

**Keywords:** P2X7 receptors, neurodegeneration, peripheral immune system, blood brain barrier, microglia

## Abstract

P2X7 receptors are ion-gated channels activated by ATP. Under pathological conditions, the extensive release of ATP induces sustained P2X7 receptor activation, culminating in induction of proinflammatory pathways with inflammasome assembly and cytokine release. These inflammatory conditions, whether occurring peripherally or in the central nervous system (CNS), increase blood-brain-barrier (BBB) permeability. Besides its well-known involvement in neurodegeneration and neuroinflammation, the P2X7 receptor may induce BBB disruption and chemotaxis of peripheral immune cells to the CNS, resulting in brain parenchyma infiltration. For instance, despite common effects on cytokine release, P2X7 receptor signaling is also associated with metalloproteinase secretion and activation, as well as migration and differentiation of T lymphocytes, monocytes and dendritic cells. Here we highlight that peripheral immune cells mediate the pathogenesis of Multiple Sclerosis and Parkinson’s and Alzheimer’s disease, mainly through T lymphocyte, neutrophil and monocyte infiltration. We propose that P2X7 receptor activation contributes to neurodegenerative disease progression beyond its known effects on the CNS. This review discusses how P2X7 receptor activation mediates responses of peripheral immune cells within the inflamed CNS, as occurring in the aforementioned diseases.

## Introduction

Purinergic receptors participate in the primordial cell signaling mechanism triggered by adenosine tri^–^ and diphosphate (ATP and ADP, respectively) and adenosine molecules. Purines are involved in several cellular functions through their binding to P1 and P2 receptors. P2 receptors are further divided into P2Y (metabotropic) and P2X (ionotropic) receptors, which include the P2X7 receptor (Burnstock, 2018), composed of two transmembrane domains and assembled as trimeric receptors. When activated, P2X receptors allow the efflux of K^+^ and influx of Na^+^ and Ca^2+^ (Bartlett et al., 2014). Besides being involved in several physiological processes, such as neurotransmitter release and proliferation, survival and activation of microglia and peripheral immune cells, P2X7 receptors are activated at extracellular ATP concentrations above homeostatic concentrations (0.05–1 mM) (Burnstock and Kennedy, 2011). The increased concentration of extracellular ATP, instead of causing desensitization of the receptor, as it occurs in other receptors, induces cell death through the opening of membrane pores. These pores increase membrane permeability to hydrophilic solutes and allow the passage of molecules of up to 900 Da which can impair homeostasis (Di Virgilio et al., 2018). ATP hydrolysis and consequent decrease in its concentration promotes the closure of these pores (Di Virgilio, 2007).

**FIGURE 1:**
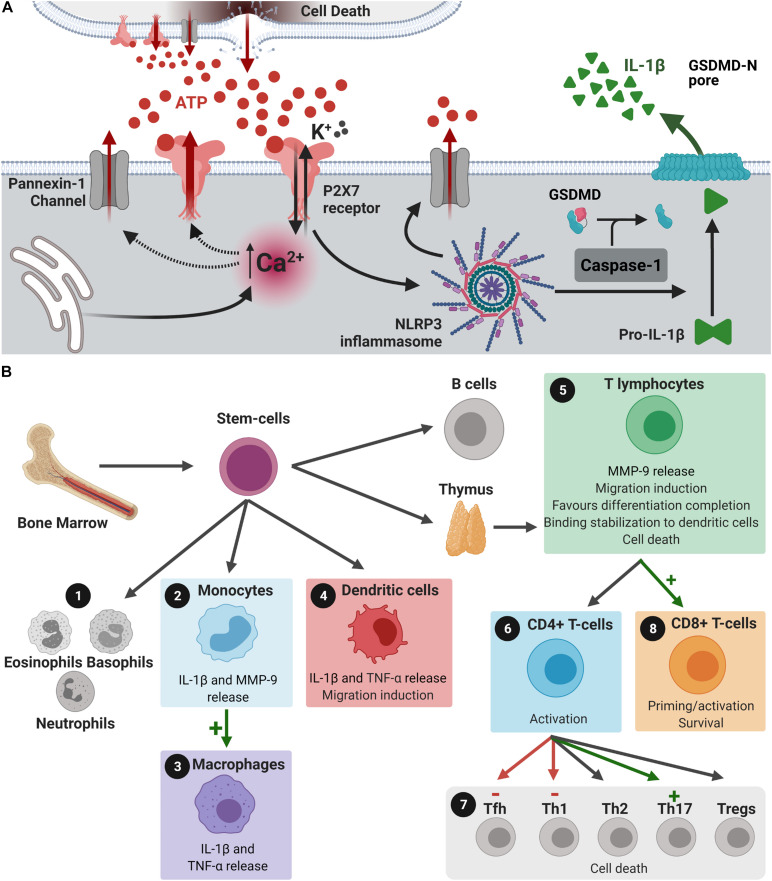
P2X7 receptors in peripheral immune cells and IL-1β release. **(A)** Dying cells release ATP. ATP binds and activates P2X7 receptors, which induces Ca^2+^ influx and K^+^ efflux. Intracellular Ca^2+^ concentration increase induces the formation of large pores by P2X7 receptors and pannexin-1, both allowing the release of more ATP. This results in augmented extracellular ATP concentration and increased activation of P2X7 receptors, providing positive feedback effects. K^+^ efflux induces NLRP3 inflammasome assembly and the formation of more pannexin-1 channels. NLRP3 inflammasome assembly activates caspase-1, which cleaves pro-IL-1β into IL-1β and promotes its release. Caspase-1 also cleaves the inhibitory gasdermin (GSDMD)-D-C into the reactive GSDMD-D-N, that interacts with membrane phospholipids to form a pore that allows IL-1β release. This release could also occur through microvesicles, exosomes or endosomes (not shown). **(B)** Bone marrow stem cells can differentiate into **(1)** eosinophils, basophils, neutrophils, **(2)** monocytes, **(4)** dendritic cells, **(5)** T and B lymphocytes. **(2)** Activation of the P2X7 receptor in monocytes induces the release of MMP-9, which can induce disruption of BBB, and IL-1β liberation. **(3,4)** P2X7 receptor activation induces the differentiation of monocytes into macrophages and stimulates the release of IL-1β and TNF-α from monocytes, macrophages, and dendritic cells. **(4)** Released IL-1β stimulates the migration of dendritic cells. **(5)** In T lymphocytes, P2X7 receptor activation may: release MMP-9, induce the migration of these cells, favor differentiation into CD4^+^ or CD8^+^ cells before leaving the thymus, stabilizes its binding to dendritic cells and promote cell death, if necessary. IL-1β released by dendritic cells through P2X7 receptor stimulation activates CD4^+^ cells and **(6)** differentiation of these cells into T helper (Th) 1 and 17, and T follicular helper cells (Tfh). **(7)** Activation of P2X7 receptors in CD8^+^ cells promotes activation and survival of these cells, while IL-1β released by dendritic cells primes CD8^+^ cells. **(8)** P2X7 receptor expression is essential for differentiation of T cells into CD8^+^ cells. Created with BioRender.com.

The P2X7 receptor presents several alternative splicing isoforms. In humans, only the A and B isoforms form functional receptors. The P2X7A is the full-length isoform and the P2X7B isoform is a truncated form. The B isoform, although assembled as a functional channel, is not able to form large pores (Adinolfi et al., 2010). P2X7 receptor activation is tightly involved in pro-inflammatory events, involving several cytokines. As illustrated in Figure 1A, P2X7 receptor activation induces K^+^ efflux and NLRP3 inflammasome assembly, cleaving pro-caspase-1 into active caspase-1. Caspase-1 is responsible for converting pro-IL-1β into mature IL-1β, cleaving the autoinhibitory domain of Gasdermin-D-C (free carboxi terminal ending), and subsequently creating its Gasdermin-D-N (free amino terminal ending) active form (He et al., 2015; Lieberman et al., 2019). Gasdermin-D-N oligomerizes upon interaction with membrane phospholipids generates a membrane pore responsible for the release of interleukins of the interleukin-1 family (IL-1), such as IL-1β and IL-18, worsening the inflammatory scenario (Liu et al., 2016). In addition to their liberation through the Gasdermin-D pore, cytokines of the IL-1 family may also be released into the extracellular space by microvesicles, exosomes or endosomes (Giuliani et al., 2017). In addition, the NLRP3/gasdermin-D pathway is also responsible for inducing membrane permeabilization and pyroptose. This pathway promotes an inflammatory type of cell death that releases intracellular content, acting as damage associated molecular pattern, exacerbating inflammatory status (Sborgi et al., 2016; Voet et al., 2019). IL-1β triggers formation of other proinflammatory factors, such as reactive oxygen species (ROS), nitric oxide (NO), and tumor necrosis factor α (TNF-α) (Giuliani et al., 2017).

ATP binding to P2X7 receptors activates the NLRP3 inflammasome, inducing cell death and tissue damage and the release of more ATP, stimulating microglial cells and recruiting peripheral immune cells. At this point, ATP release/P2X7 receptor activation/apoptosis axis act on a positive feedback loop, greatly contributing to the neurodegenerative process in a continuous manner. Immune cells release more cytokines and chemokines, exacerbating the inflammatory scenario and activating pro-apoptotic cascades culminating in cell death. Furthermore, P2X7 receptor activation increases glutamate secretion, which could aggravate glutamate excitotoxicity observed in neurodegenerative diseases (Spérlagh et al., 2002; Cho et al., 2010). The neuroinflammatory process observed in neurodegenerative diseases is mediated by cells of the peripheral immune system and by resident microglial cells (Prinz and Priller, 2017). Neuroinflammation broadly refers to immune processes taking place in the central nervous system (CNS) that are elicited by a stimulus, such as infection, physiological stress or neurological disease, among others. The released proinflammatory factors induce increased blood-brain barrier (BBB) permeability and facilitate peripheral immune cells neuroinvasion (Prinz and Priller, 2017). A temporary stimulus tends to be beneficial, but if it persists, it may affect CNS homeostasis, synaptic transmission, and plasticity. This seems to be the case for neurodegenerative diseases, where continuous activation of immune cells promotes exacerbated inflammation, leading to cognitive decline (Lee et al., 2010). The entire process that culminates in the neuroinvasion of peripheral immune cells, as well as the neuroinflammation itself, has stages modulated by the P2X7 receptor (Figure 1B). The participation of the P2X7 receptor in innate immune responses stimulating the inflammasome in the peripheral immune system (Ratajczak et al., 2020), as well as in the brain contributing to neuroinflammation and neuropsychiatric diseases (Ratajczak et al., 2019) has been proposed.

This review provides evidence for the participation of the P2X7 receptor in neuroinflammatory processes mediated by peripheral and resident immune cells. While roles of P2X7 receptors in neuroinflammation and neurodegeneration have been extensively discussed in the literature, the current knowledge of possible involvement of these receptors in neuroinvasion by peripheral immune cells is scarce.

## Neuroinvasion of Peripheral Immune Cells: Blood-Brain-Barrier Permeability

The BBB is a highly selective barrier that allows the communication between the CNS and the peripheral system, regulating molecule transport into and from the brain and leading to a fine tune modulation of microenvironment homeostasis (Zlokovic, 2011; Zhao et al., 2015; Montagne et al., 2017). Its major functions include entry control of pathogens, blood cells and neurotoxic components from the bloodstream into the CNS. The BBB protects the CNS from peripheral circulation and is composed of endothelial cells (ECs), astrocytes and pericytes that are linked via different connections, such as tight junctions, tricellular junctions and adherent junctions. The interplay between these cells is fundamental to BBB function. The set of morphological and molecular interactions between these cells is called neurovascular unit (NVU), which conducts the maintenance of CNS homeostasis through intercellular signaling and physical communication (Iadecola, 2017; Sweeney et al., 2019).

Cerebral ECs present tight junction proteins bonded to the cytoskeleton, such as claudin-5 and occludin, which participate in paracellular communication processes of BBB with distinct functions (Engelhardt and Liebner, 2014; Anderson and Van Itallie, 1995). The disruption in claudin-5 leads to increased BBB permeability and high mortality in mice, and occludin disruption induces brain calcification (Saitou et al., 2000; Nitta et al., 2001). Destabilization of BBB cellular organization can be found in cerebral injuries (Shlosberg et al., 2010) caused by increased levels of inflammatory cytokines and ROS (Gasche et al., 2001; Katsu et al., 2010). These proinflammatory factors activate matrix metalloproteinases (MMPs) and zinc–calcium dependent endopeptidases, which can degrade proteins of the extracellular matrix and tight junctions in the ECs (Vecil et al., 2000; Liu and Rosenberg, 2005; Katsu et al., 2010; Gottschall, 2016).

**FIGURE 2:**
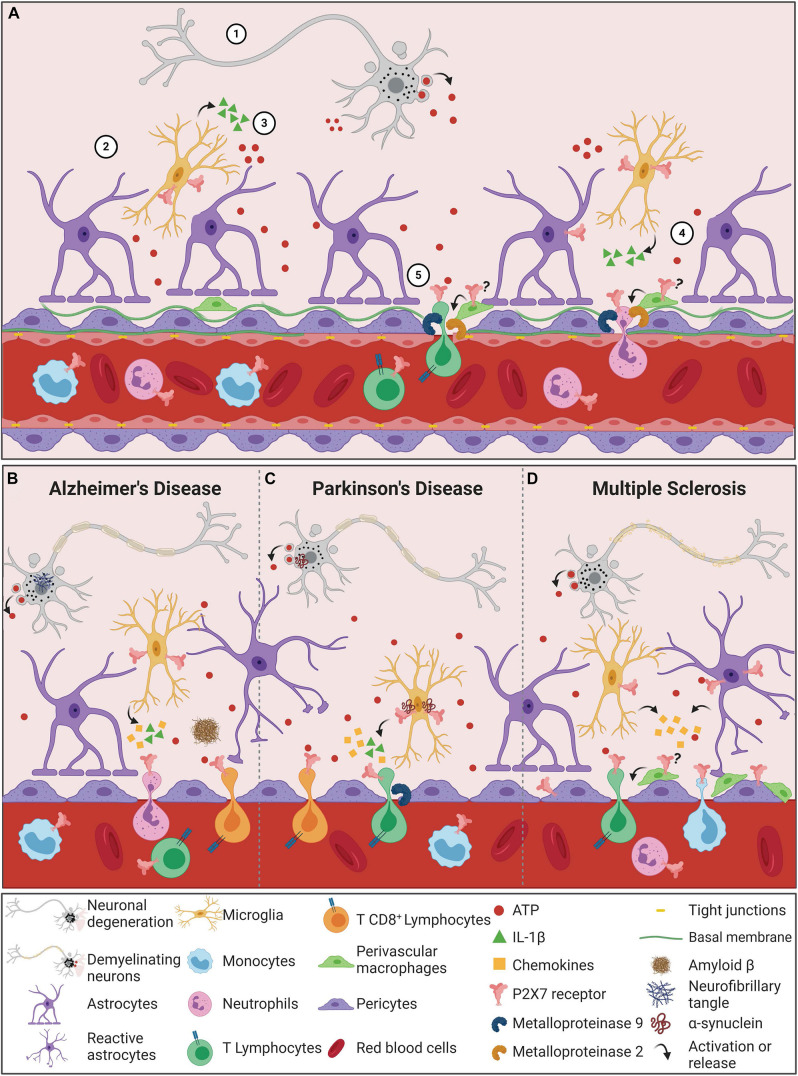
The P2X7 receptor in the process of Blood-Brain Barrier permeabilization during neurodegenerative diseases. **(A)** Blood-Brain Barrier permeabilization through P2X7 receptor signaling begins with cellular damage, which triggers cell death and consequently ATP release into the CNS microenvironment **(1)**. ATP content above physiologic ranges activates P2X7 receptors in microglial cells **(2)**, which in turn leads to the production and release of IL-1β and other cytokines and chemokines **(3)**. Moreover, the active ATP/P2X7 receptor pathway augments metalloproteinase (MMP) activities, causing TJ and BM protein degradation and consequently BBB destabilization **(4)**. The outcome of these combined events is the peripheral immune cell invasion of the CNS. Since T cells and neutrophils express P2X7 receptors, the amount of ATP released by the damaged cells can also activate this receptor in these cells. The result is the shedding activation of adhesion proteins and the production and release of cytokines and MMPs, promoting an inflammatory and invasive feedback mechanism **(5)**. In neurodegenerative diseases, the neuronal death process induces the release of high concentrations of ATP into the extracellular milieu. **(B)** In AD pathogenesis, ATP binds to P2X7 receptors that are overexpressed in microglial cells. The microglial activation depends on P2X7 receptor expression/activity to react against Aβ peptides. P2X7 receptor stimulates the release of IL-1β and chemokines that contribute to increased BBB permeability, resulting in chemotaxis of neutrophil and CD8^+^ cells. **(C)** In PD, α-synuclein aggregates contribute to dopaminergic neuronal death, increasing ATP levels and P2X7 overactivation. α-synuclein can bind to P2X7 receptors expressed in microglial cells. Both α-synuclein and P2X7 receptors are associated with increased release of IL-1β and chemokines, indicating an enhanced neuroinflammatory scenario in PD. This IL-1β secretion is associated with induction of MMP-9 release, resulting in BBB dysfunction. Under condition of BBB dysfunction and increased chemokine levels, T cells are chemoattracted and infiltrate brain parenchyma. T cells can recognize α-synuclein epitopes, release high amounts of ATP and present upregulation of P2X7 receptor activity, contributing to PD worsening. **(D)** The demyelinating character of MS is also associated with increased ATP levels and P2X7 receptor overactivation. P2X7 receptor expression and activity are upregulated in reactive astrocytes and contribute to the release of chemokines and peripheral immune cell chemotaxis. Pericytes also express P2X7 receptors that mediate the interaction between endothelial cells and pericytes and contribute to BBB integrity. Increased numbers of perivascular macrophages are associated with disease progression, and P2X7 receptor functions in this process remain unknown. MS exhibits infiltrating T cells and monocytes, expressing P2X7 receptors that contribute to the autoimmune attack and disease progression. (?) Hypothesis of P2X7 receptor involvement in perivascular macrophages, yet to be elucidated. Created with BioRender.com.

Proposed roles of ATP/P2X7 signaling in tight junction impairment processes are presented in Figure 2A. The release of IL-1β following P2X7 receptor activation triggers the production of MMP-9, degrading occludins and disrupting BBB integrity, as observed in human and rat *in vitro* models, as well as *in vivo* in mice (Harkness et al., 2000; Mori et al., 2002; Gu and Wiley, 2006; Rosenberg, 2009). Similar results were observed in human EC and astrocyte co-culture, simulating the BBB *in vitro*. Treatment with the P2X7 receptor agonist BzATP caused significant increase in IL-1β and MMP-9 levels, resulting in decreased concentrations of tight junction proteins, such as occludin (Yang et al., 2016). These results indicate that the degradation of tight junction proteins induced by ATP/P2X7 receptor signaling is strongly associated with IL-1β receptors, which plays a fundamental role in BBB dysfunction (Yang et al., 2016).

Pericytes are the other cell type essential for optimal BBB formation, function and maintenance (Bell et al., 2010; Daneman et al., 2010; Nisancioglu et al., 2010). They alter EC behavior through activity modulation of transporters and expression of ion channels and junction proteins (Rucker et al., 2000; Franco et al., 2011; Bai et al., 2015). ECs and pericytes are the cells from the NVU with the strongest contact, interacting through peg-and-socket junctions. This interaction allows the transport of nutrients and metabolic signaling to the CNS. Both cell types are inserted in the basement membrane, with proteins that are crucial to BBB integrity and stability. The basement membrane is a unique form of extracellular matrix, known as the BBB non-cellular component. It comprises four major types of proteins: laminin, nidogen, perlecan, and collagen IV (Nisancioglu et al., 2010; Baeten and Akassoglou, 2011). Involvement of the P2X7 receptor in degradation of collagen IV by the neurotoxic compound 3,4-methyllenedioxymethamphetamine (MDMA) was demonstrated in rats. Administration of MDMA increased levels of P2X7 receptor expression in activated microglia cells. This effect was accompanied by enhanced proteolytic activities of hippocampal MMP-3 and MMP-9, resulting in laminin and collagen IV degradation (Figure 2A; Rubio-Araiz et al., 2014). Associated with these results, leakage of IgG into the brain parenchyma was found, which indicates higher BBB permeability. Remarkably, treatment with Brilliant Blue G (BBG, P2X7 receptor antagonist), prevented P2X7 receptor expression increase in rat hippocampus and subsequent effects observed by MDMA administration (Rubio-Araiz et al., 2014). Thus, that data suggests that P2X7 receptor activation increases BBB permeability triggered by MDMA through stimulation of MMPs. Recently, the immune system has been suggested as a new target of pericytes. Pericytes can modulate phagocytosis, adhesion molecules expression, stimulate neutrophil transmigration and generation of proinflammatory mediators, as inducible nitric oxide synthase (iNOS), ROS and cyclooxygenase (COX). Thus, it is suggested that pericytes participate in neuroinflammation processes (Hurtado-Alvarado et al., 2014; Pieper et al., 2014; Wang and Sanz, 2016; Rustenhoven et al., 2017).

Astrocytes are also important for BBB maintenance. The astrocyte end-foot covers ECs and communicates with them through gap and peg-and-socket junctions (Kim et al., 2009; Bonkowski et al., 2011; Winkler et al., 2011). They are a source of signaling molecules that regulate NVU functioning (Engelhardt and Liebner, 2014). Rats submitted to intracerebral hemorrhage presented increased P2X7 receptor expression in astrocytes and ECs. This scenario was succeeded by neuronal death, neurobehavioral and neurofunctional deficits, cerebral edema, increased RhoA (known to regulate the formation of adhesions and stress fibers) expression and a decreased expression of structural endothelial proteins, such as occludin and vascular endothelial cadherin (Mueller, 2014). Treatment with A438079, a selective P2X7 receptor inhibitor, or P2X7 receptor gene knockdown reduced these phenotypes. Additionally, this treatment also prevented parenchymal leakage induced by intracerebral hemorrhage (Zhao et al., 2016).

The BBB is also composed of perivascular macrophages (PVMs), which are resident CNS macrophages located in the perivascular space (or Virchow-Robin space). Despite being under debate, PVMs seem to originate from the yolk sac similarly to microglial cells, although they are distinguished by the expression of specific proteins (Bechmann et al., 2001; Goldmann et al., 2016). The exact role of brain PVMs in the BBB permeability remains unknown, but their phagocytic activity selectively restricts the crossing of serum proteins and macromolecules into the brain parenchyma (Willis et al., 2007). Additionally, PVMs have an important role protecting the brain during bacterial and viral infections (Polfliet et al., 2001; Filipowicz et al., 2016). These cells connect the peripheral immune system with the CNS through the regulation of neutrophil (Polfliet et al., 2001), T cell (Tran et al., 1998) and leukocyte infiltration by MMP-2 and MMP-9 activity modulation (Figure 2A; Agrawal et al., 2006; Bechmann et al., 2007). Under conditions of systemic immune activation, PVMs regulate the hypothalamo-pituitary-adrenal (HPA) axis, prostaglandin expression, controlling inflammation in rat and mouse models (Serrats et al., 2010; Vasilache et al., 2015). These features are relevant for CNS diseases. In fact, it is hypothesized that PVMs may exert protective effects in the initial phases of CNS diseases, avoiding disease progression and misfolded protein spreading; however, repeated PVM activation might result in detrimental and progressive effects of CNS diseases (Koizumi et al., 2019). P2X7 receptor expression in macrophages and its role in inflammation and immunity is widely recognized, as discussed below. Noteworthy, P2X7 receptor stimulation of the NLRP3 inflammasome pathway is essential in macrophage polarization (Di Virgilio et al., 2017; Savio et al., 2018). However, the P2X7 receptor was not yet investigated in PVMs, evidencing a relevant gap in the field. Considering the control of inflammation by PVMs, the surveillance of immune infiltrating cells, the modulation of macrophage polarization and the role of the P2X7 receptor in CNS diseases, we propose that P2X7 receptors regulate the physiological and pathological functions of PVMs, assisting BBB permeability shifts.

Relevant for this topic, the infiltration of peripheral immune cells through the BBB is a core feature of its disruption. Interactions between adhesion proteins expressed by ECs, integrins and selectins expressed by peripheral cells are necessary for infiltration (Greenwood et al., 2002; Nisancioglu et al., 2010). It is known that trans endothelial migration of peripheral immune cells depends on various factors, such as the shedding of CD62L and CD21 membrane proteins. Brain injuries cause cellular damage and, consequently, higher levels of extracellular ATP (Volonte et al., 2003; Melani et al., 2005). This event is often associated with increased BBB permeability and invasion of peripheral cells in the CNS. Thus, the participation of P2X7 as a mediator in this *trans* endothelial migration signaling pathway has been investigated. It has been suggested that P2X7 receptor mediates the participation of ROS and extracellular ATP in the induction of MMPs activity, causing CD62L shedding in B lymphocytes (Foster et al., 2013). Similar assays using treatments with P2X7 receptor agonists and antagonists confirmed that induction of CD62L and CD21 shedding in T cells, B cells and neutrophils are mediated by the P2X7 receptor (Jamieson et al., 1996; Gu et al., 1998; Sengstake et al., 2006). Altogether, these data propose a different pathway by which the P2X7 receptor modulates immune functions in an indirect way, having as target the peripheral immune cells that penetrate the CNS toward injury areas.

## Peripheral Immune Cells

The peripheral immune system is composed of lymphocytes, including T-, B-, and natural killer cells, neutrophils and monocytes/macrophages (Figure 1B). During CNS injury, peripheral innate (monocytes) and adaptative (B and T) immune cells can infiltrate the CNS according to the extent of the BBB permeability (Prinz and Priller, 2017). The P2X7 receptor is suggested to be involved in several actions exerted by these cells (Figure 1B). For instance, a single nucleotide polymorphism in the P2X7 receptor gene (Glu496Ala) that induces loss-of-function was identified in humans. This polymorphism protected whole blood samples against cytotoxic effects of high concentrations of ATP (Wesselius et al., 2012) and reduced IL-1β release by monocytes (Sluyter et al., 2004). Additionally, P2X7 receptor activation induced the release of MMP-9 by peripheral blood mononuclear cells and in both purified monocyte and T-cell populations (Gu and Wiley, 2006), indicating that immune cells may release factors that also contribute to BBB disruption, favoring brain invasion. Dendritic cells, responsible for activating T-cells by positioning them in the surface of cells in contact with pathogens, migrate and release interleukins and pro-inflammatory factors upon P2X7 receptor activation, determining T-cell polarization (Rivas-Yáñez et al., 2020). Once in the brain, these factors could increase neuroinflammation, worsening brain injury in neurodegenerative diseases.

### Monocytes

In the healthy brain, monocytes reside in the endovascular space and do not invade brain parenchyma (Prinz and Priller, 2017). However, in the diseased brain, these cells migrate to injured areas and differentiate into macrophages with high phagocytic and inflammatory activities, which in some cases may result in disease worsening and autoimmune attack (Yamasaki et al., 2014). Although there is no change in P2X7 receptor gene expression in the differentiation process, the amount of surface labeled P2X7 receptors increase (Gudipaty et al., 2001). As recently shown by single-cell RT-PCR, 90% of human monocyte-derived macrophages present P2X7 receptor gene expression. Sixty nine% of these cells present ATP-evoked P2X7 receptor currents (Vargas-Martínez et al., 2020).

P2X7 receptor activation in monocyte/macrophages is involved in pro-inflammatory effects. Activation of monocytic P2X7 receptors induces NLRP3 inflammasome assembly and interleukin secretion in M1 macrophages (Gu and Wiley, 2018). Interestingly, studies suggest that pore formation mediated by P2X7 receptors is independent of pannexin-1 actions (Alberto et al., 2013). However, anti-inflammatory effects of the P2X7 receptor were proposed in M2 macrophages, based on release induction of molecules involved in inflammation resolution (De Torre-Minguela et al., 2016). Moreover, in these cells, modulation of phagocytic activity by the P2X7 receptor and its beneficial effects at physiological conditions are under investigation. It is proposed that, in the presence of ATP at low concentrations, the macrophage P2X7 receptor acts as a scavenger receptor, recognizing and engulfing apoptotic cells (Gu and Wiley, 2018; Zumerle et al., 2019). This process occurs due to the formation of a complex between the P2 X 7 receptor and non-muscle myosin IIA (expressed by resting macrophage), which directly binds to the extracellular domain of apoptotic cells. The presence of high ATP concentrations promotes dissociation of this complex and inhibits phagocytic activity (Gu et al., 2010). Increased P2X7 receptor expression in human macrophages was correlated with rapid bead engulfment *in vitro*, impaired by blocking P2X7 receptor expression (Gu et al., 2011). Upon increased ATP levels, P2X7 receptor activation inhibits this phagocytic activity and exerts pro-inflammatory effects (Wiley and Gu, 2012; Gu and Wiley, 2018). Altogether, the P2X7 receptor plays essential roles in monocyte/macrophage differentiation, activation/polarization and function, both in physiological and pathological conditions.

### T Cells

T cells are a subtype of lymphocytes and comprehend CD4^+^helper and CD8^+^ cytotoxic T lymphocytes, which belong to the adaptive immune system. T helper CD4^+^ cells drive the immune response according to the pathogen (Korn and Kallies, 2017) and express both P2X7A and P2X7B receptors variants (Cheewatrakoolpong et al., 2005). As recently reviewed by Grassi (2020) and by Rivas-Yáñez et al. (2020), P2X7 receptors participate in T lymphocyte cell activation, migration, and differentiation. It interferes in lymph node traffic through CD62L shedding, thus being essential for CD4^+^ cell responses to injury (Yip et al., 2009; Foster et al., 2013; Grassi, 2020; Rivas-Yáñez et al., 2020). In the brain, as observed in the mouse model of experimental autoimmune encephalomyelitis (EAE), Th1 cells could induce macrophage and microglia cell activation through secretion of IFN γ (Murphy et al., 2010). Neuroinvasion of Th1 cells could worsen cell death, both by increasing the inflammatory processes and cytokine release from active microglia cells and by inducing demyelination.

CD8^+^ T cells recognize MHC Class I molecules, acting in defense against pathogens, such as bacteria and viruses, and promote cell apoptosis by releasing cytokine enzymes such as perforin and granzymes (Janeway et al., 2001). P2X7 receptor ablation reduces differentiation of T cells into CD8^+^ cells, inducing metabolic dysfunction and impairing CD8^+^ cells survival and function (Borges Da Silva et al., 2018). Moreover, long-term survival of CD8^+^ cells depends on a constant efflux of ATP maintained by P2X7 receptor activation, favoring their survival (Wanhainen et al., 2019). In view of that, P2X7 receptor inhibition could induce neuroprotection by reducing the number of CD8^+^ cells, which modulate synaptic plasticity and contribute to neuronal dysfunction.

Noteworthy, data regarding T lymphocytes in commercial available P2X7 receptor knockout (KO) mice should be carefully analyzed carefully. The GlaxoSmithKline strain failed to ablate P2X7 receptor variant K (P2X7K). This isoform is widely expressed in T lymphocytes, and these KO animals present increased P2X7 receptor-mediated responses in these cells (Bartlett et al., 2014).

## Microglia: Resident Immune Cells in the CNS Parenchyma

Microglial cells are the resident immune cells within the CNS. These bone-marrow independent resident phagocytes are involved in several physiologic and pathologic processes (Huang et al., 2018). In mice, these cells are involved in synaptic pruning since postnatal brain development (Paolicelli et al., 2011) and in phagocytosing apoptotic cells during zebrafish retina development (Blume et al., 2020). Within the mouse adult brain, they are involved in immune surveillance through dynamic extending cellular processes and phagocytosis of cellular debris (Eyo et al., 2014; Cserép et al., 2020).

Besides the importance of microglial cells in normal brain physiology, there is evidence for their role during onset and progression of psychiatric and neurodegenerative diseases (Bachiller et al., 2018; Tay et al., 2018). It is known that, during neurodegenerative disease development, microglia cells shift their reactive phenotypes. Classically, microglia cells *in vitro* shift from protective M2 to pro-apoptotic and inflammatory M1 phenotypes (Tang and Le, 2016). These categories follow the criteria designed for M1/M2 macrophages after cytokine release stimulation by Th1/Th2 CD4^+^ T lymphocytes, respectively (Muraille et al., 2014). However, the classification of *in vivo* reactive states is still under debate, since unbiased gene expression profile analysis failed to identify microglial M1/M2 activation states. Thus, microglial identification through classical markers might not be optimal (Ransohoff, 2016). In this sense, recent findings suggest that microglia cells indeed show a hybrid state of activation in neurodegenerative diseases, called disease-associated microglia (Keren-Shaul et al., 2017). This state is proposed to delay the initial stage of disease progression, but might present deleterious effects in patient health at later stages (Song and Colonna, 2018).

In this context, mouse and rat *in vitro* models indicated that the P2X7 receptor activation may be involved in microglial proliferation, activation, and production of TNF-α, and chemokines CCL2, CCL3, and CXCL2 (Suzuki et al., 2004; Bianco et al., 2006; Kataoka et al., 2009; Monif et al., 2009; Shiratori et al., 2010; Shieh et al., 2014). As previously discussed, P2X7 receptor activation enables the assembly of the NLRP3 inflammasome, and maturation and release of IL-1β (Bianco et al., 2005; Xu et al., 2020), triggering neuronal death (Kaur et al., 2014; Stojakovic et al., 2017) in rat and murine cell lines. This deleterious effect is supported by *in vivo* studies. Mice lacking NLRP3 or the IL-1 receptor (which binds IL-1β) showed fewer neurodegenerative features after sepsis-induced LPS challenges (Zhao et al., 2020). Moreover, upon stimulation by LPS, striatal microglial cells turn to the activated profile and started to produce several cytokines, inducing P2X7 receptor expression upregulation. In this study, the pharmacological modulation with oxidized ATP, an P2X7 receptor antagonist, diminished the LPS effect and prevented neuroinflammation in rats (Choi et al., 2007). Controversially, murine microglia *in vitro* stimulated with a potent P2X7 receptor agonist showed downregulation of microglial P2X7 receptor expression. This treatment induced a remarkable loss of cell viability of microglia *in vivo* after LPS and/or IFN-γ stimulation (He et al., 2017), highlighting alternative P2X7 receptor activity. Thus, pre-clinical evidence supports P2X7 receptor participation in microglia driving neuroinflammation.

Considering the close relationship between P2X7 receptors and microglial cells, initial studies in humans proposed that P2X7 receptor ligands coupled to radiotracers can address the neuroinflammatory status in subjects suffering from neuroinflammatory diseases. The authors suggest that this technique would better diagnose neuroinflammation than commonly used markers do. However, a larger cohort is necessary before applying this alternative for clinical use (Hagens et al., 2020). On the other hand, another in-human pilot study with 10 Parkinson’s Disease (PD) volunteers failed to identify differences in P2X7 receptor radiotracer incorporation between healthy and PD subjects (Van Weehaeghe et al., 2019). In this sense, P2X7 receptor roles in triggering microglial activation could be independent from its overexpression. However, this depends on alternative P2X7 receptor functioning, such as macropore formation (Van Weehaeghe et al., 2019). Although radioligand administration did not reveal any risk for healthy subjects, the efficacy of targeting P2X7 receptors as diagnostic tools for the assessment of microglial inflammation status still requires further elucidation (Van Weehaeghe et al., 2019). Thus, microglial proliferation and activation, as well as the release of pro-inflammatory factors, contribute to neuroinflammatory and neurodegenerative scenarios, augmenting the release of ATP and activating even more P2X7 receptors, worsening neuroinflammation and neurodegeneration.

## P2X7 Receptor Immune Modulation as a Target for Neurodegenerative Diseases

In physiological conditions, the P2X7 receptor presents low expression within CNS cells. In contrast, during stress situations in which extracellular ATP reaches high concentrations, the expression of the P2X7 receptor increases. Hence, the use of pharmacological inhibition as a therapeutic strategy seems interesting from the homeostasis point of view, considering that broad P2X7 receptor activation occurs mostly in inflammatory conditions, such as neurodegenerative diseases (Figure 2B). These diseases include PD, Alzheimer’s disease (AD), and Multiple Sclerosis (MS). The mechanisms underlying neurodegenerative process are yet to be elucidated, although it is known that aging and neuroinflammation are key aspects (Stephenson et al., 2018). A growing number of studies indicate that not only CNS resident cells are associated with neurodegeneration. Peripheral immune cells also seem to have a key role. As previously discussed, P2X7 receptor expression is abundant in immune cells, both CNS resident and peripheral ones. Strong evidence points at beneficial effects of P2X7 receptor antagonism in diseases with neuroinflammation as a culprit of neurodegeneration (Andrejew et al., 2020). For instance, P2X7 receptor inhibition was recently proposed to reduce neuronal complications related to the SARS-CoV-2 infection in patients. In this scenario, the exacerbated inflammatory response induced by SARS-CoV-2 in the lungs might be mediated by P2X7 receptor activation in lung and immune cells, inducing the ‘cytokine storm.’ This process increases BBB permeability, allowing neuroinvasion by infected peripheral immune cells and SARS-CoV-2 infection of CNS cells. In agreement with that, infiltration of such cells into the CNS may result in neuroinflammation, neurodegeneration and consequent neuropsychiatric symptoms, which are widely reported by COVID-19 patients (Ribeiro et al., 2021). Thus, it has been proposed that P2X7 receptor inhibition functions as a therapeutic strategy to dampen neuroinflammation, consequently decreasing neurodegeneration, especially in AD, PD and MS.

### Alzheimer’s Disease

Alzheimer’s Disease (AD) is a neurodegenerative disorder characterized by the formation of insoluble protein deposits in the CNS, composed by amyloid β (Aβ) plaques and phosphorylated Tau tangles (Polanco et al., 2018). These aggregates trigger a strong inflammatory response promoted by microglia and astrocyte activation, which are the major source of cytokines and chemokines in AD (Heneka et al., 2015), allowing the infiltration of peripheral immune cells that enhance neuroinflammation (Figure 2B; Rossi et al., 2011). Although the literature provides compelling evidence for P2X7 receptor-induced effects on microglia during AD pathology, it lacks studies regarding the direct action of this receptor on peripheral immune cells. The infiltration of leukocytes is commonly observed in *postmortem* samples of AD subjects (Togo et al., 2002). Indeed, neutrophil CNS infiltration is associated with AD progression and contributes to cognitive decline in animal models of AD (Zenaro et al., 2015). These data reinforce the importance of studying P2X7 receptors known to modulate immune responses on peripheral immune cells and their neuroinvasion process.

P2X7 receptor roles in resident CNS immune cells (i.e., microglia) are well established, as discussed above. Pharmacological P2X7 receptor inhibition is strongly considered as a therapeutic approach in AD, mainly because its critical role on NLRP3-induced IL-1β release by microglia (Biber et al., 2019). Activation of microglia in response to Aβ peptides requires P2X7 receptor expression. This was confirmed by the lack of IL-1β release through inflammasome activation in cultured P2X7 receptor KO microglia and P2X7 receptor KO mice (Sanz et al., 2009). Microglia samples obtained *postmortem* from AD subjects displayed a higher expression of P2X7 receptors, when compared to healthy individuals, confirming microglia liability of P2X7 receptor activation (McLarnon et al., 2006; Martin et al., 2019).

Microglial phagocytic activity helps the CNS to perform ideal embryonic development and maintain adult homeostasis. Thus, it has come to attention that the impairment of this function is associated with neurodegenerative diseases (Galloway et al., 2019). Genome wide association studies indicated strong links between microglial genes and AD risk (Salter and Stevens, 2017). Aβ plaque formation, the main hypothesis for AD development, focuses on the impairment of microglial activity on Aβ clearance (Hardy and Higgins, 1992). In addition, phagocytosis by peripheral monocytes is also important in AD. Vascular system monocytes are attracted to the walls of Aβ-positive veins, promoting Aβ elimination (Michaud et al., 2013). Fresh monocytes isolated from healthy individuals and AD patients displayed normal basal phagocytic activity. However, monocytes derived from patients with increased Aβ burden had their phagocytic index activity inhibited by ATP (Gu et al., 2016). As mentioned before, P2X7 receptor expression by microglia and monocytes grant these cells a phagocytic ability in the absence of ATP, acting as a scavenger receptor (Gu and Wiley, 2018). Thus, high levels of ATP, as observed in exacerbated inflammation and neurodegeneration scenarios, inhibit P2X7-mediated phagocytosis (Gu et al., 2011). Considering that phagocytosis has a key role in AD pathogenesis, these data indicate that P2X7 receptor scavenger activity may be important for Aβ clearance and preventing disease progression.

While the NLRP3 has an important participation in neuroinflammation, P2X7 receptor signaling can also provide additional immune responses, as its deficiency reduces brain chemokine production. Chemokines are small proteins important for chemotaxis, recruiting peripheral immune cells. In cell culture and mouse models of AD, brain expression of chemokines is upregulated in response to Aβ aggregation. In a mouse AD model, KO for the P2X7 receptor resulted in differential expression of chemokines. APPPS1xP2X7 KO mice presented significantly lower cerebral levels of chemokines CCL3, CCL4, CCL5, when compared to regular APPPS1 mice (Martin et al., 2019). Since the main function of chemokines is leukocyte recruitment, this result indicates that the lack of P2X7 receptor activation can result in decreased brain chemotaxis. CCL3 and CCL4 (MP1-α and MP1-β, respectively) and CCL5 chemokines are increased in brains of AD subjects (Tripathy et al., 2010), suggesting that pharmacological inhibition of the P2X7 receptor could decrease this pro-inflammatory effect during AD pathology. Further, the APPPS1xP2X7 KO mouse also displayed significantly lower infiltration of CD8^+^ T cells, both in the hippocampus and choroid plexus (Martin et al., 2019). These results confirm the specific role of the P2X7 receptor in immune responses observed during AD, since its deficiency reduced brain chemokine release and T cell infiltration. In this regard, several patents of brain-penetrant P2X7 receptor antagonists were already proposed, including one for AD treatment (Bhattacharya, 2018).

### Parkinson’s Disease

Parkinson’s disease (PD) is a neurodegenerative disease characterized by death of dopaminergic neurons from the nigrostriatal pathway and accumulation of α-synuclein aggregates (Marino et al., 2019). An increased number of reactive microglia cells with phagocytic activity was found in postmortem brains of PD subjects (Toulorge et al., 2016), evidencing the tight relation between neuroinflammation and neurodegeneration observed in this disease (Oliveira-Giacomelli et al., 2020). In a rat model of PD, increased microglial activation is observed in injured striatum (Carmo et al., 2014) and substantia nigra, accompanied by increased P2X7 receptor gene expression (Oliveira-Giacomelli et al., 2019). This effect was prevented by the use of the P2X7 receptor antagonist BBG, enabling dopaminergic neuron recovery (Oliveira-Giacomelli et al., 2019). The neuroregeneration observed in the presence of P2X7 receptor antagonists is likely to be mediated by inhibition of microglial activation, since nigral dopaminergic neurons lack P2X7 receptor expression (Marcellino et al., 2010). In fact, a recent study showed that ligand-P2X7 receptor binding is increased in the striatum and substantia nigra of 6-OHDA lesioned rats and P2X7 receptor expression is mainly associated with microglial cells (Crabbé et al., 2019). Corroborating this hypothesis, IL-1β released from microglia cells supposedly augments susceptibility of substantia nigra dopaminergic neurons to cell death in an animal model of PD (Koprich et al., 2008). Expression of the P2X7 receptor is upregulated in the brain of PD subjects and in 6-OHDA injured rats (Durrenberger et al., 2012; Oliveira-Giacomelli et al., 2019), which agrees with the hypothesis that microglial hyperactivation and consequent neuroinflammation contribute to dopaminergic neurodegeneration. Finally, extracellular α-synuclein was shown to activate murine microglial cells *in vitro* through P2X7 receptor activation and oxidative stress exacerbation (Jiang et al., 2015).

α-synuclein protein, a key component of PD pathogenesis, binds to P2X7 receptors in microglia and stimulates its transcription (Jiang et al., 2015). Some studies found that PD subjects can have circulating T cells that are capable of recognizing specific epitopes of α-synuclein, indicating that PD might result from an autoimmune response (Sulzer et al., 2017). These α-synuclein reactive T cells may be detected years before the appearance of motor symptoms, indicating a possible target for early diagnosis of PD (Lindestam Arlehamn et al., 2020). Non-human primate PD model and PD subjects displayed chronic brain infiltration of T cells (Galiano-Landeira et al., 2020; Seo et al., 2020). Once in the brain, T cells recognizing α-synuclein proteins undergo activation and initiate processes that recruit P2X7 receptors.

Purinergic signaling is a critical component for the amplification of T cell receptor signaling (Junger, 2011). Following antigen-recognition, T cells release a large amount of ATP through pannexin channels, which can bind and activate P2X7 receptors. Indeed, activation of T cell receptors upregulated P2X7 expression in purified CD4^+^ T human cells (Yip et al., 2009). Blockade of P2X7 receptors through different approaches, such as the use of specific antagonists, addition of apyrase to hydrolyze extracellular ATP or silencing P2X7 with siRNA, inhibited T cell activation (Yip et al., 2009). In PD subjects, the presence of brain infiltrated CD8^+^ T cells was higher compared to control subjects, and their density positively correlated with neuronal death (Galiano-Landeira et al., 2020). Considering the liability of P2X7 receptor activation on T cell function, the T cell/P2X7 axis is an important matter in PD pathology (Figure 2C).

Chronic brain infiltration by T cells and other peripheral immune cells only occurs due to BBB disruption, which is commonly observed in neurodegenerative diseases (Sweeney et al., 2019). In PD, the aggregation of α-synuclein was seen to be related to BBB dysfunction. Addition of α-synuclein significantly increased the permeability of ECs in rat brain ECs co-cultured with rat brain pericytes. This increase depended, at least in part, on pericytes activation. In pericyte culture alone, α-synuclein induced cytokine release, such as IL-1β, IL-6, TNF-α, monocyte-attractant chemokine MCP-1 and MMP-9 (Dohgu et al., 2019). Interestingly, P2X7 receptor activation induces IL-1β release, which in turn promotes MMP-9 secretion and consequent BBB disruption (Yang et al., 2016). A transgenic mouse model overexpressing human α-synuclein, revealed impaired BBB integrity, with vascular dysfunction, decreased levels of collagen IV and activation of pericytes, even at early stages (Elabi et al., 2021). *In vivo* models already showed that microglia activation in response to a neurotoxic compound induces expression upregulation of P2X7 receptors, accompanied by increased MMP-9 activity and collagen IV degradation (Rubio-Araiz et al., 2014). Importantly, α-synuclein-induced microglial BV2 cell line activation is critically mediated by P2X7 receptors, leading to neuroblastoma SH-SY5Y cell apoptosis (Jiang et al., 2015). These results strongly associate P2X7 receptor activity with resident and peripheral immune cell activation during PD pathology.

### Multiple Sclerosis

Several autoimmune diseases have been already associated with P2X7 receptor activation, including lupus erythematosus, rheumatoid arthritis, Sjögren’s syndrome, systemic sclerosis, inflammatory bowel syndrome and Multiple Sclerosis (MS) (Cao et al., 2019). MS is an autoimmune inflammatory disorder characterized by glial cell activation, loss of oligodendrocytes, immune cell axonal damage and demyelination, mostly influenced by infiltrated peripheral immune cells (Pinheiro et al., 2016). *Postmortem* samples of MS subjects revealed increased expression of P2X7 receptors by reactive astrocytes, especially in the frontal cortex parenchyma (Narcisse et al., 2005; Amadio et al., 2017). Nevertheless, CNS inflammation observed in MS is not only due to resident immune cell activation. Neuroinflammation observed in MS and experimental model EAE, the most common rodent model used for studying inflammation and demyelination in MS, is a consequence of a strong infiltration of peripheral immune cells.

Studies investigating the P2X7 receptor in the EAE mouse model revealed conflicting results. The absence of P2X7 receptor in mice seemed to decrease astroglial activation, an effect that was associated with a reduced incidence of EAE (Sharp et al., 2008). In this direction, Bernal-Chico et al. (2020) aimed to study the role of P2X7 receptor KO in primary demyelination and remyelination, using the cuprizone model of T-cell independent myelin degeneration. Their results agree with protective features in the absence of P2X7 receptor expression. In response to cuprizone, P2X7 deficient mice presented diminished M1 microglia and astrogliosis, mitigated demyelination and decreased expression of pro-inflammatory genes (Bernal-Chico et al., 2020). On the other hand, reports suggest that P2X7 KO mice develop more severe clinical and pathological expression of EAE. This detrimental effect would occur through mechanisms that involve a loss of apoptotic activity in lymphocytes (Chen and Brosnan, 2006) and the enhancement of axonal damage when developing EAE (Witting et al., 2006). It is important to highlight that the diverging results can be explained, at least in part, by P2X7 receptor KO models applied in these studies. At least two mouse strains with P2X7 receptor knockout are available for research purposes. Sharp and collaborators, who observed a beneficial effect of the P2X7 KO (Sharp et al., 2008) used the GlaxoSmithKline strain, in which the P2X7A receptor isoform is absent, while the P2X7K receptor isoform is still expressed. These mice display increased P2X7 receptor-mediated responses in T-cells (Chessel et al., 2005). In contrast, works describing a prejudicial effect of P2X7 receptor KO (Chen and Brosnan, 2006; Witting et al., 2006) used the Pfizer strain (Solle et al., 2001). This strain is deficient in both P2X7A and P2X7K isoforms of the receptor. The P2X7A and P2X7K isoforms seem to have unique features. For example, the P2X7K isoform is more sensitive for activation by agonists. Lower concentration of BzATP activates the P2X7K isoform, as determined in the ethidium bromide uptake assay. Further, P2X7K receptor isoform induced-ethidium uptake was independent from pannexin-1 action (Xu et al., 2012). In the P2X7A receptor isoform, the receptor C-terminal segment interacts with pannexin-1, inducing the formation of large pores in the cell membrane and leading to apoptosis (Masin et al., 2012). Therefore, it is important to establish the differences reported in the literature for each strain, for better understanding of involved mechanisms and results.

Infiltrated myelin-specific T cells stimulate neuroinflammation and BBB dysfunction (Figure 2D; Yamasaki et al., 2014). During MS progression, brain infiltration of leukocytes has a major contribution to the neuroinflammation observed in this pathology. MS has two hypotheses regarding the infiltration of peripheral immune cells in the CNS. The “outside-in” hypothesis indicates that CD4^+^ T cells are activated in the periphery and then cross the BBB. Once in the CNS, they can recognize other antigens and initiate a pro-inflammatory cascade, promoting neuroinflammation. Oppositely, the “inside-out” hypothesizes that MS is originally a neurodegenerative disease eventually triggering an autoimmune reaction (Trapp and Nave, 2008). As mentioned before, P2X7 receptors exert principal functions in the signaling cascade that follows T cell activation (Yip et al., 2009). Despite the evidence that P2X7 receptors are widely expressed in both hematopoietic-derived and CNS resident immune cells (Collo et al., 1997; Yiangou et al., 2006; Hanley et al., 2012), knowledge is scarce regarding P2X7 receptor signaling in immune cells during inflammation processes of MS pathology. It is known that the KO of the P2X7 gene in mice leads to a more severe clinical and pathological EAE (Chen and Brosnan, 2006). Circulating monocytes of healthy subjects express the P2X7 receptor, and this expression was observed to be downregulated in monocytes obtained from stable and acute MS subjects. The same result was identified in the rat EAE model. On the other hand, infiltrated monocytes expressing P2X7 receptors were also upregulated in the frontal cortex blood vessels of secondary progressive MS subjects. In the same area of the frontal cortex, increased expression of the leukocyte-attractant chemokine MCP-1, was co-localized with P2X7 receptor expression (Amadio et al., 2017). MCP-1 is also co-localized with astrocytes in MS-derived lesions, as shown in human *postmortem* tissue (Simpson et al., 1998). Further, *in vitro* assays revealed that BzATP through P2X7 receptor activation enhanced MCP-1 expression in rat astrocytes (Panenka et al., 2001). Amadio et al. (2017) developed the hypothesis that during initial stages of MS, CNS homeostasis loss leads to an increase in extracellular ATP, activating P2X7 receptors, which are upregulated in their expression levels in astrocytes. Increased release of MCP-1 promotes peripheral monocyte chemotaxis, which in turn downregulates P2X7 receptor expression to ensure their survival and CNS invasion. The result is augmented neuroinflammation, resulting in neurodegeneration.

The MS EAE mouse model is also linked to increased BBB permeability (Alvarez et al., 2011). P2X7 receptor expression was found in pericytes of cerebral microvessels in both EAE and wild-type (WT) rats. In the EAE model, ATP-dependent P2X7 receptor activation induced oligodendrocyte excitotoxicity and promoted neuronal damage similar to that observed in MS, including demyelination and axonal impairment (Matute et al., 2007). Upregulation of P2X7 receptor expression located in astroglia of these animals can be observed at early stages, before symptoms, such as tail paralysis and loss of reflexes, have developed (Grygorowicz et al., 2010). EAE rats develop neurological deficits, whose severity was reduced when BBG had been administered. Throughout the development of the disease, the animals showed increased P2X7 receptor expression, followed by decreased pericyte immunostaining and claudin-5 levels (Grygorowicz et al., 2018). Treatment with the P2X7 receptor antagonist BBG restored pericyte and claudin-5 levels (Grygorowicz et al., 2018). As pericytes are responsible for proper regulation of BBB permeability, their deficit leads to a reduction in tight junction proteins (Bell et al., 2010) and to an increase in BBB permeability (Nisancioglu et al., 2010). Thus, these results indicate the participation of the P2X7 receptor as a mediator of interactions between ECs and pericytes in MS. Evidence suggests direct and indirect participation of this receptor in the processes that cause BBB rupture and permeabilization in some neurological disorders. However, exact mechanisms need to be better elucidated for the understanding of the molecular biology of the disorders and especially for possible therapeutic approaches.

Due to the autoimmune nature of MS and its effects on BBB integrity, PVMs are a promising target in MS. In fact, PVMs seem to contribute to MS progression and pathogenesis. Increased numbers of PVMs were detected in MS brain subjects (Zhang et al., 2011) and in the EAE animal model, as well as CD163-associated PVMs expression and leukocyte infiltration before symptoms’ onset (Polfliet et al., 2002). PVM depletion induced by clodronate liposomes protected against symptoms’ appearance (Polfliet et al., 2002). Additionally, spinal PVMs presented upregulation of CD45, CD106, CCL2, and CCL3 protein expression in the EAE model (Hofmann et al., 2002), indicating that chemokines may recruit monocytes and contribute with the demyelinating pathogenesis. The leukocyte chemoattractant CCL3 chemokine is also released by ECs during inflammation (Chui and Dorovini-Zis, 2010). Importantly, in an AD neuroinflammation model, the KO of the P2X7 receptor significantly decreased the CCL3 chemokine release (Martin et al., 2019), indicating that the activation of this receptor may contribute to this inflammatory aspect of MS.

## Conclusion

The neurodegenerative process is often accompanied by an exacerbated neuroinflammatory response, with increased ATP release, microglia proliferation, as well as activation and release of proinflammatory factors. These processes are known to be mediated by P2X7 receptors, through assembly of NLRP3 inflammasome and maturation/secretion of IL-1β, TNF-α and other cytokines and chemokines. In turn, IL-1β triggers production of MMP-9 by immune and BBB cells, degrading tight junction proteins and resulting in BBB disruption.

The permeable BBB allows the infiltration of peripheral immune cells into the CNS. The availability of these cells involves P2X7 receptor activation, since it modulates differentiation, release, activation and function of monocytes/macrophages and T cells. Moreover, although promising, there are no available data regarding P2X7 receptor modulation of PVMs activity, since it regulates peripheral immune cells trafficking through the BBB. Once in the brain, these cells can also induce microglial responses. Basically, P2X7 receptor involvement in these processes occurs as a positive feedback loop, releasing factors that amplify its activation, resulting in exacerbation of the inflammation due to microglial activation, immune cells recruitment, and autoimmune responses with consequent worsening of neurodegeneration. In neurodegenerative disorders, the initial inflammatory process is crucial for disease progression and is highly upregulated by P2X7 receptor activation. In AD, PD, and MS, microglial activation and leukocyte infiltration are immune features observed in the brain. This proinflammatory state occurs in early disease stages and in parallel to disease progression, as a key factor leading to demyelination and neuronal death. Thus, P2X7 receptor antagonism could be a promising target for neurodegenerative diseases associated with inflammation and immune responses in the brain.

## Author Contributions

ÁO-G: sections “Neuroinvasion of Peripheral Immune Cells: Blood-Brain-Barrier Permeability” and “P2X7 Receptor Immune Modulation as a Target for Neurodegenerative Diseases” writing and Figure 1. LP: section “P2X7 Receptor Immune Modulation as a Target for Neurodegenerative Diseases” writing and manuscript organization. RA: sections “Peripheral Immune cells” and “Conclusion” writing and Figure 2. NT: section “Peripheral Immune Cells” writing and Figure 2. JS: sections “Conclusion” and “Microglia” writing and Figure 2. US: manuscript conception and critical article review for important intellectual content. HU: manuscript conception and writing and critical article review for intellectual content. All authors contributed to the article and approved the submitted version.

## Conflict of Interest

The authors declare that the research was conducted in the absence of any commercial or financial relationships that could be construed as a potential conflict of interest.

## Abbreviations

AD: Alzheimer’s disease
ATP: adenosine triphosphate
BBB: blood-brain barrier
BBG: Brilliant Blue G
CD4: cluster of differentiation 4
CD8: cluster of differentiation 8
CD62L, cluster of differentiation 62 L: also known as L-selectin
CCL3: chemokine (C-C motif) ligand 3
CNS: central nervous system
EAE: experimental autoimmune encephalomyelitis
ECs: endothelial cells
IL-1 β: interleukin 1 β
LPS: lipopolysaccharide
MCP-1: monocyte chemoattractant protein-1
MDMA: 3,4-methyllenedioxymethamphetamine
MS: multiple sclerosis
MMP-9: matrix metalloproteinases 9
NLRP3: NOD-like receptor protein 3
NO: nitric oxide
PD: Parkinson’s disease
ROS: reactive oxygen species
TNF- α: tumor necrosis factor α.
